# MiR-26a-5p/EZH2 Mediates Wnt2 Promoter Methylation to Regulate Trophoblast Dysfunction

**DOI:** 10.2174/0113862073360368250404104831

**Published:** 2026-04-24

**Authors:** Xiaoyu Zhou, Shiqi Wei, Ning Yu, Yufang Liu

**Affiliations:** 1 Department of Gynaecology and Obstetrics, Binzhou Medical University Hospital, Binzhou, Shandong 256600, China;; 2 Department of Pathology, Binzhou Medical University Hospital, Binzhou, Shandong 256600, China

**Keywords:** miRNA, Wingless-type MMTV integration site family, member 2, preeclampsia, methylation, apoptosis, inflammation, methyltransferase

## Abstract

**Introduction:**

Preeclampsia (PE) is a common complication of pregnancy, with a concomitant incidence rate of up to 10% among pregnant women worldwide.

**Methods:**

In the current research, we explored the role and mechanism of miR-26a-5p in trophoblast function using CCK-8, colony formation assay, and flow cytometry. The interaction between miR-26a-5p and EZH2 was analyzed using a luciferase reporter assay. Methylation-specific PCR was performed to detect the methylation level of Wnt2 in HTR8 cells.

**Results:**

Wnt2 and miR-26a-5p promoted the proliferation and inhibited the apoptosis in trophoblasts (*P<*0.05). The secretion of inflammatory cytokines was suppressed by Wnt2 and miR-26a-5p (*P<*0.05). EZH2 was identified as a regulatory target of miR-26a-5p using HTR8 cells and bioinformatic tools. miR-26a-5p inhibited expression through direct binding to EZH2. Importantly, miR-26a-5p mediated DNA methylation of Wnt2 to regulate Wnt2 expression in HTR8 cells.

**Discussion:**

This study elucidates a novel regulatory axis that alleviates trophoblast dysfunction by promoting proliferation and suppressing inflammation and apoptosis. The findings reveal that the miR-26a-5p/EZH2/Wnt2 pathway, potentially involving promoter methylation, is crucial for maintaining trophoblast function. This work identifies a promising therapeutic target for PE, although further *in vivo* validation is required to confirm its clinical potential.

**Conclusion:**

It was found that miR-26a-5p increased the expression of Wnt2 by downregulating EZH2. Moreover, miR-26a-5p/EZH2/Wnt2 promoted the proliferation and inhibited the inflammation and apoptosis in trophoblasts. This research provides insight into the role of miR-26a-5p/EZH2/Wnt2 as a novel indicator for the prevention and treatment of PE.

## 1 INTRODUCTION

Preeclampsia (PE) is a common complication of pregnancy, with a concomitant incidence rate of up to 10% among pregnant women worldwide. PE is one of the main reasons for the increase in maternal and perinatal mortality [1, 2]. Research has confirmed that abnormal trophoblast cell function is a crucial factor in PE pathogenesis [3].

MicroRNAs (miRNAs) are highly conserved short, noncoding RNAs that affect disease progression by regulating the levels of genes and proteins [4]. miR-26a-5p plays an important role in inflammation and tumors of reproductive systems [5-8]. miR-26a-5p has been identified in extracellular vesicles during pregnancy and is associated with PE and miscarriage [9]. miR-26a can promote endothelial cell proliferation, migration, and tube formation through glycolysis in early-onset preeclampsia (EOPE) [10]. Although miR-26a-5p is associated with PE progression, its specific role in trophoblast cells remains unclear.

In the present research, we explored the role of miR-26a-5p in HTR8 cells and identified Wnt2 as a downstream target. Wnt2 is an important member of the Wnt family, which activates downstream genes through the Wnt/β-catenin pathway and regulates physiological and pathological processes [11, 12]. Previous studies indicated a close correlation between Wnt2 and PE pathogenesis [13]. Our study explored the molecular mechanism by which miR-26a-5p regulated Wnt2 expression, thereby providing potential targets for the prevention and treatment of PE.

## 2 MATERIALS AND METHODS

### 2.1 Cell Culture and Transfection

Human chorionic trophoblast cell line HTR8 was purchased from the American Type Culture Collection and cultured in DMEM supplemented with 10 % FBS at 37 °C with 5 % CO_2_. Cell transfection (50 ng sequences or plasmids) was conducted using Lipo2000 (Invitrogen, Carlsbad, CA, USA) according to the manufacturer’s manual.

### 2.2 Cell Counting Kit-8 (CCK-8)

CCK-8 assay was performed to analyze cell viability. HTR8 cells were seeded in a 96-well plate at 2000 cells/well. Then, 10 μL of 5 mg/mL CCK-8 (MedChemExpress, Monmouth Junction, NJ, USA) was added into wells and incubated for 1 h. The optical density value was detected at 450 nm using a microplate reader.

### 2.3 Colony Formation Assay

After 24 h of transfection, HTR8 cells were seeded into 60 mm dishes at a density of 500 cells/dish and cultured for 2 weeks. Then, cells were fixed with 4 % paraformaldehyde for 30 min and stained with 0.1 % crystal violet for 5 min. The colony number was counted.

### 2.4 ELISA

After 24 h of transfection, cell supernatant was collected for the detection of inflammatory factors. ELISA kits for IL-1β, IL-6, and TNF-α were purchased from ABclonal Biotechnology (Woburn, MA, USA). ELISA was performed according to the manufacturer’s instructions. In brief, samples were incubated with the HRP-labeled detection antibodies for 1 h, and the optical density was analyzed at 450 nm on a plate reader.

### 2.5 Flow Cytometry

The apoptosis of HTR8 cells was analyzed using an Annexin V-FITC/PI kit. Cells were resuspended in a cold binding buffer and transferred to an EP tube. Cells were then incubated with 5 μl of staining solution at room temperature in the dark for 5 min. The PI staining solution (10 μL) was then added. Cell apoptosis was detected using flow cytometry, and the data were analyzed using FlowJo software.

### 2.6 Western Blot

Cells were lysed using RIPA buffer supplemented with phosphatase inhibitor after transfection for 24 h. Samples were separated by SDS-PAGE and transferred to a PVDF membrane. After blocking with 5% non-fat milk, proteins were incubated at 4°C overnight with the primary antibodies and then incubated with secondary antibodies for 2 h. The bands were visualized using an ECL-PLUS kit (Amersham Pharmacia Biotech, Piscataway, NJ, USA). The primary antibodies anti-BCL-2 (ABclonal, Woburn, MA, USA), anti-BAX (Proteintech, Chicago, IL, USA), anti-Cleaved-Caspase 3 (Affinity, Jiangsu, China), anti-β-Catenin (Proteintech, Chicago, IL, USA), anti-P-GSK3B (Cell Signaling Technology, Inc., Beverly, MA, USA), anti-GSK3B (Proteintech, Chicago, IL, USA), and anti-GAPDH (Proteintech, Chicago, IL, USA) were used in the research.

### 2.7 Luciferase Reporter Assay

A luciferase reporter assay was performed to analyze the interaction between miR-26a-5p and enhancer of zeste 2 polycomb repressive complex 2 subunit (EZH2). Briefly, cells were co-transfected with miR-26a-5p mimics and a wild/mutant EZH2 plasmid. After transfection for 48 h, cells were lysed. Dual-Luciferase Reporter Assay System (Promega, Madison, WI, USA) was used for the detection of luciferase activity. Then, samples were analyzed using a Varioskan LUX microplate reader (Thermo Scientific, San Jose, CA, USA).

### 2.8 Quantitative Real-time PCR (qRT-PCR)

RNA was extracted from HTR8 cells using TRIzol reagent (CWBio, Beijing, China). Reverse transcription and qRT-PCR were performed using the HiFiScript cDNA synthesis kit (CWBio, Beijing, China) and UltraSYBR mixture (CWBio, Beijing, China), respectively. The following primers used in this research were synthesized from Genewiz (Suzhou, China): Wnt2, forward 5'-AAGAAGATGG GAAGCGCCAA-3' and reverse 5'-GGCTCTGGCATCCTTT CCTT-3'; EZH2, forward 5'-CCCGCTGAGGATGTGGAT AC-3' and reverse 5'-CATGGTTAGAGGAGCCGTCC-3'; β-actin, forward 5'-AGACCTGTACGCCAACACAG-3' and reverse 5'-CGGACTCGTCATACTCCTGC-3'. The relative expression level of the target gene was analyzed using the 2^-∆∆ Ct^ method.

### 2.9 Methylation-specific PCR (MSP)

After 24 h of transfection, DNA was extracted from HTR8 cells. PCR was performed to detect methylation levels. Primers in this research were as follows: M-WNT2(M)-F, 5'-AGTTGCGTTTGGAGGGGAAC-3'; M-WNT2(M)-R, 5'-CTACATCAAATCAAACTCCGTATACG-3'; M-WNT2 (U)-F, 5'-TTTAGTTGTGTTTGGAGGGGAAT-3'; M-WN T2(U)-R, 5'-CTACATCAAATCAAACTCCATATACA-3'.

### 2.10 Statistical Analysis

Results were obtained from three independent experiments. Data were analyzed using GraphPad Prism software (version 9.0). The ANOVA method was used for the comparison of cells in each group. Statistical significance was set at *P <* 0.05. All data were expressed as the mean ± SD.

## 3 RESULTS

### 3.1 Wnt2 and miR-26a-5p Promoted the Proliferation in Trophoblasts

To investigate the role of Wnt2 and miR-26a-5p in trophoblasts, Wnt2 siRNA, Wnt2 expression plasmid, miR-26a-5p, and miR-26a-5p inhibitor were transfected into HTR8 cells. First, we detected the proliferation of each group of cells using CCK-8 and colony formation assays. As shown in Fig. (**1A**), Wnt2 knockdown inhibited the proliferation, whereas Wnt2 overexpression promoted the proliferation of HTR8 cells. Cell viability increased significantly after the transfection with miR-26a-5p and declined significantly after the transfection of miR-26a-5p inhibitor. In colony formation assay, Wnt2 knockdown and miR-26a-5p inhibitor markedly decreased the colony number, while Wnt2 overexpression and miR-26a-5p significantly increased the colony number in HTR8 cells (Fig. **1B**). These results proved that Wnt2 and miR-26a-5p promoted trophoblast proliferation.

### 3.2 Wnt2 and miR-26a-5p Inhibited the Secretion of Inflammatory Cytokines In Trophoblasts

In this study, we also detected the levels of inflammatory cytokines IL-1β, IL-6, and TNFα in cell supernatant (Figs. **1C**-**E**). Wnt2 knockdown significantly increased the levels of IL-1β, IL-6, and TNFα, which were markedly attenuated by Wnt2 overexpression. miR-26a-5p significantly promoted the expression of IL-1β, IL-6, and TNFα, while miR-26a-5p inhibitor significantly reduced the expression of IL-1β, IL-6, and TNFα in HTR8 cells. These results indicated that Wnt2 and miR-26a-5p inhibited the secretion of inflammatory cytokines in trophoblasts.

### 3.3 Wnt2 and miR-26a-5p Inhibited the Apoptosis in Trophoblasts

The role of Wnt2 and miR-26a-5p on apoptosis in trophoblasts was examined using flow cytometry and FlowJo software (Fig. **2A**). Flow cytometry results proved that the percentage of apoptotic cells increased after Wnt2 knockdown but decreased after Wnt2 overexpression compared with that in the CON group. miR-26a-5p significantly declined the percentage of apoptotic cells, which were markedly increased by miR-26a-5p inhibitor. To clarify the mechanism by which Wnt2 and miR-26a-5p inhibited apoptosis, we detected the expression of apoptosis-related proteins in trophoblasts. As shown in Figs (**2B**-**E**), Wnt2 and miR-26a-5p inhibitors attenuated the expression of apoptosis inhibitor Bcl-2 and promoted the expression of cleaved-caspase 3 and Bax. In contrast, Wnt2 overexpression and miR-26a-5p enhanced Bcl-2 expression and inhibited the expression of cleaved-caspase 3 and Bax in HTR8 cells.

### 3.4 miR-26a-5p Inhibited EZH2 Expression through Direct Binding to EZH2

TargetScan database was used to analyze the target gene of miR-26a-5p [14]. As demonstrated by the data analyzed, EZH2 contained potential binding sites for miR-26a-5p (Fig. **3A**). To demonstrate the relationship between miR-26a-5p and EZH2, the wild/mutant type of EZH2 plasmids were constructed and transfected into cells by co-transfection with miR-26a-5p mimics. As shown in (Fig. **3B**), fluorescence intensity significantly declined in cells co-transfected with wild-type (WT) EZH2 plasmid and miR-26a-5p mimics compared with cells transfected with only miR-26a-5p mimics. Mutant type (MUT) EZH2 plasmid did not demonstrate this effect. This result proved that miR-26a-5p bound directly to the 3’ UTR of EZH2 through the predicted site. Then, qPCR results demonstrated that miR-26a-5p mimics significantly inhibited the expression of EZH2 in HTR8 cells (Fig. **3C**). The protein level of EZH2 was suppressed by miR-26a-5p mimics and promoted by miR-26a-5p inhibitors (Figs. **3D** and **E**).

### 3.5 miR-26a-5p Mediated DNA Methylation of Wnt2

EZH2 performs methyltransferase function in the genome [15]. Research reports that the methylation of the Wnt2 promoter is significantly associated with PE progression [16, 17]. Here, we investigated the effects of miR-26a-5p and EZH2 on Wnt2 promoter methylation in HTR8 cells. As shown by the MSP results, Wnt2 exhibited a high level of methylation in control cells (Fig. **4**). After transfection with miR-26a-5p, the methylation level of EZH2 significantly declined, suggesting that miR-26a-5p mediated DNA methylation of Wnt2 to enhance Wnt2 transcription. Interestingly, EZH2 overexpression also inhibited the methylation level of EZH2 in HTR8 cells. We hypothesized that the regulation ofWnt2 methylation by miR-26a-5p/EZH2 is influenced by other factors, requiring further investigation.

### 3.6 miR-26a-5p/EZH2 Regulated the Expression of Wnt2 in HTR8 Cells

We then detected the expression of Wnt2 in HTR8 cells transfected with miR-26a-5p mimics or inhibitors. As shown in Fig. (**5A**), Wnt2 expression was promoted by miR-26a-5p and inhibited by an miR-26a-5p inhibitor. To further confirm the regulatory effect of miR-26a-5p/EZH2 on Wnt2, HTR8 cells were transfected with miR-26a-5p, EZH2 expression plasmid, or co-transfected with miR-26a-5p and EZH2 expression plasmid (Fig. **5B**). Wnt2 mRNA level increased significantly in EZH2 overexpression cells and declined significantly in co-transfected cells in comparison to the EZH2 overexpression cells (Fig. **5C**). Consistently, the protein level of Wnt2 was upregulated by EZH2 overexpression compared with the control cells and downregulated in co-transfected cells compared with the EZH2 overexpression cells (Figs. **5D**-**F**).

## 4 DISCUSSION

Previous studies have confirmed that abnormally expressed miRNAs can lead to placental dysplasia and pregnancy diseases [18-20]. Research works focusing on miRNAs reveal the pathogenesis of PE and are expected to explore new targets for the prediction, treatment, and evaluation of PE [21, 22]. miR-26a-5p is a relatively conserved member of the miRNA family, widely expressed in various tissues and cell types [23-26]. miR-26a-5p is upregulated in PE placental tissue, regulating the proliferation, invasion, and migration of trophoblast cells by binding to the 3 'untranslated region of N-cadherin [27, 28]. *In vivo* studies have reported that miR-26a-5p stimulates the proliferation of porcine trophoblasts and affects early pregnancy by regulating gene expression [29]. In the present research, we demonstrated that miR-26a-5p promoted proliferation and inhibited the secretion of inflammatory cytokines and apoptosis in trophoblasts. miR-26a-5p alleviated trophoblast damage by regulating cell proliferation and apoptosis and has potential preventive and therapeutic effects on human PE.

miRNAs regulate the occurrence and development of diseases through the regulation of downstream targets [30-32]. In this study, we investigated the downstream mechanism by which miR-26a-5p alleviated trophoblast dysfunction. Wnt2 was found to enhance the proliferation and inflammatory cytokine levels and suppress the apoptosis in HTR8 cells. The crucial role of Wnt2 in PE has been reported in several studies [16]. Mice with low Wnt2 exhibited placental damage, including decreased fetal capillaries and increased fibrinoid deposition, as well as placental phenotype abnormalities associated with poor placental angiogenesis [33, 34]. The inhibition of Wnt2 weakened the proliferation and migration of trophoblast cells, thereby reducing trophoblast invasiveness and shallow placental implantation [35].

Importantly, miR-26a-5p and Wnt2 not only regulated the trophoblast cell function but also inhibited the inflammatory response. Patients with PE exhibited a significant increase in various inflammatory factors in peripheral blood, which is an abnormal chronic inflammatory state. Inhibiting inflammation, reducing the overexpression of inflammatory factors, or decreasing the activity of inflammatory activation pathways may reduce the risk of PE. This evidence suggests that miR-26a-5p and Wnt2 are potential targets for PE prevention and treatment.

Bioinformatics analysis results demonstrated that EZH2 was a binding target for miR-26a-5p, and luciferase reporter assay proved the direct combination between EZH2 3’UTR and miR-26a-5p. Then, qPCR and western blot results demonstrated that miR-26a-5p upregulated Wnt2 expression by inhibiting EZH2 in trophoblast cells. EZH2 is a regulatory factor in PE progression. Targeting EZH2 affects the proliferation, migration, and tube formation in trophoblasts [36, 37].

EZH2 is a member of the Polycomb-group (PcG) family and a catalytic subunit of Polycomb Repressive Complex 2 (PRC2) [38]. It functions as a histone H3K27 methyltransferase [39]. Shang *et al.* confirmed that the inhibition of EZH2 in trophoblasts promotes polarization of M1 macrophages, leading to recurrent spontaneous abortion [40]. Combining our findings, EZH2 may be an important regulatory factor for functional abnormalities in trophoblasts.

Many studies have provided strong evidence that the pathogenesis of PE is closely related to Wnt2 methylation status [41, 42]. Methylation data from GSE44712 indicates that Wnt2 has been identified as hypermethylation in the PE placenta [43]. In humans, Wnt2 is highly expressed during placental development [44, 45]. Methylation of the Wnt2 promoter is significantly associated with a decrease in Wnt2 expression in the placenta and is inversely proportional to newborn birth weight and Cole index [17, 43, 46]. We further investigated whether miR-26a-5p/EZH2 regulated Wnt2 transcription through methylation. Overexpression of miR-26a-5p and EZH2 led to the downregulation of the methylation ratio of the Wnt2 promoter. Although the results were unexpected, miR-26a-5p/EZH2 altered the methylation level of the Wnt2 promoter. However, the regulatory mechanism of miR-26a-5p/EZH2/Wnt2 requires further investigation. Due to off-target effects, gene inhibitors or siRNA may unexpectedly affect other non-target cells, causing side effects in clinical applications, which is also a major limiting factor for most technologies or drugs in tumor treatment. Therefore, more experiments are still needed. This research investigated the effect of miR-26a-5p/EZH2/Wnt2 in trophoblasts *via* a cell model. However, this functional axis was not validated in an *in vivo* model. Therefore, further investigation is still needed to confirm the results of the present research.

## CONCLUSION

In this study, it was found that miR-26a-5p promoted the expression of Wnt2 *via* downregulating EZH2. miR-26a-5p/EZH2/Wnt2 promoted the proliferation and inhibited the inflammation and apoptosis in trophoblasts. This research provides insights into the role of miR-26a-5p/EZH2/Wnt2 as a novel indication for the prevention and treatment of PE.

## STUDY LIMITATIONS

This study has several limitations. Firstly, the lack of validation in animal models or primary human placental tissues limits the understanding of this regulatory axis within the complex physiological context of a whole organism. Additionally, the precise molecular mechanism by which the miR-26a-5p/EZH2 axis regulates Wnt2 transcription requires further elucidation. Future studies employing *in vivo* models and clinical samples are essential to confirm the pathophysiological relevance and therapeutic potential of the miR-26a-5p/EZH2/Wnt2 pathway in PE.

## AUTHORS' CONTRIBUTIONS

The authors confirm their contribution to the paper as follows: XZ is responsible for investigation and writing original draft. SW, and NY assisted in the experiment, formal analysis and data curation. YL is responsible for methodology, editing and funding acquisition. All authors reviewed the results and approved the final version of the manuscript.

## Figures and Tables

**Fig. (1):**
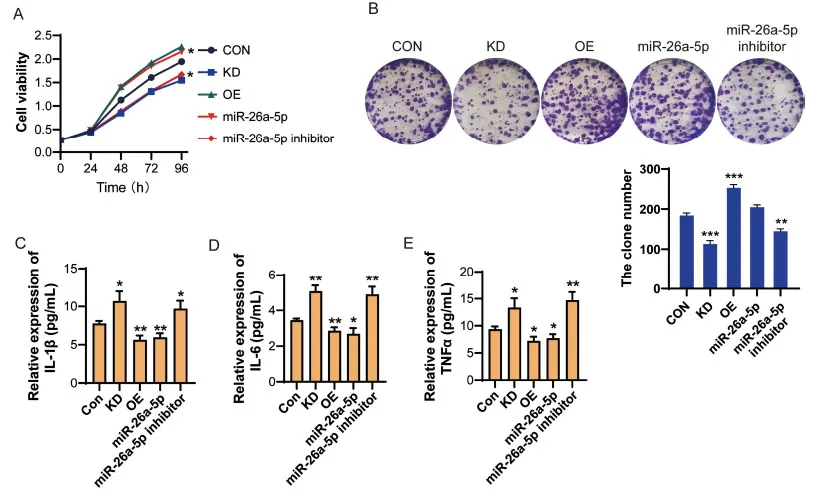
Wnt2 and miR-26a-5p promoted the proliferation and inhibited the secretion of inflammatory cytokines in trophoblasts. (**A**) Wnt2 siRNA (KD), Wnt2 expression plasmid (OE), miR-26a-5p, and miR-26a-5p inhibitor were transfected into HTR8 cells. Cell viability was detected every 24 h using a CCK8 reagent. (**B**) The proliferation was confirmed using a colony formation assay. (**C**) IL-1β, (**D**) IL-6, and (**E**) TNFα were detected using ELISA. * *P<*0.05 *vs*. con, ** *P<*0.01 *vs*. con, *** *P<*0.001 *vs*. con.

**Fig. (2):**
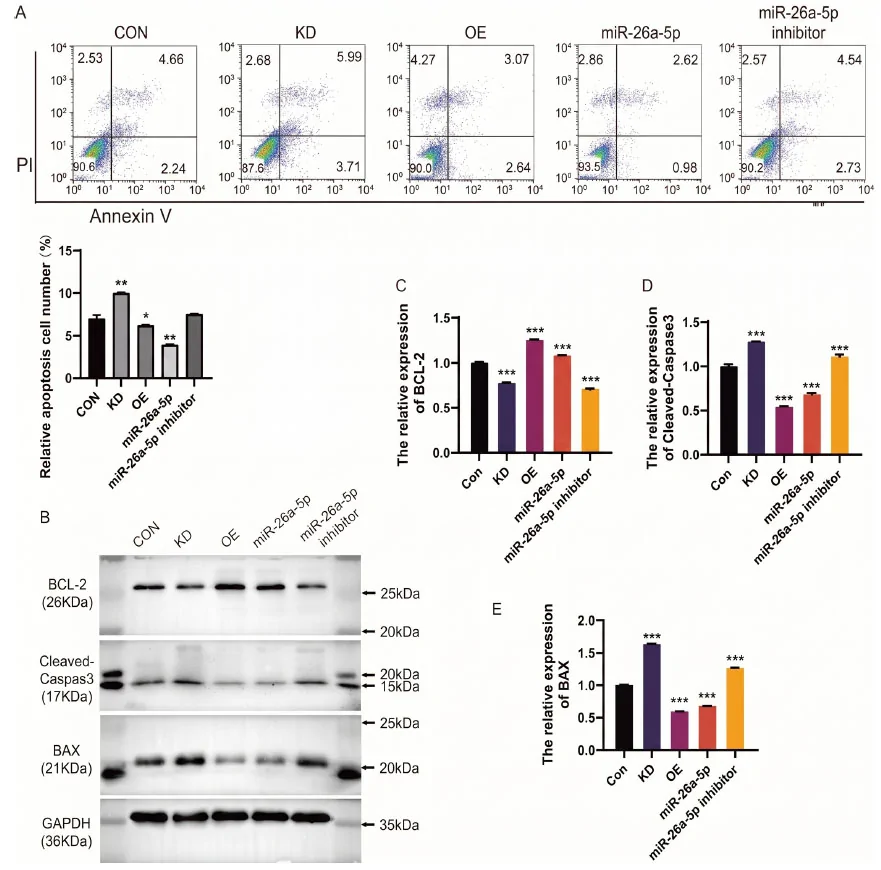
Wnt2 and miR-26a-5p inhibited the apoptosis in trophoblasts. (**A**) Cell apoptosis was analyzed using flow cytometry. (**B**) Apoptosis-related protein expressions were confirmed by western blot. (**C**) Relative expression levels of Bcl-2, (**D**) cleaved-caspase 3, and (**E**) Bax were analyzed using ImageJ software. * *P<*0.05, ** *P<*0.01, *** *P<*0.001.

**Fig. (3):**
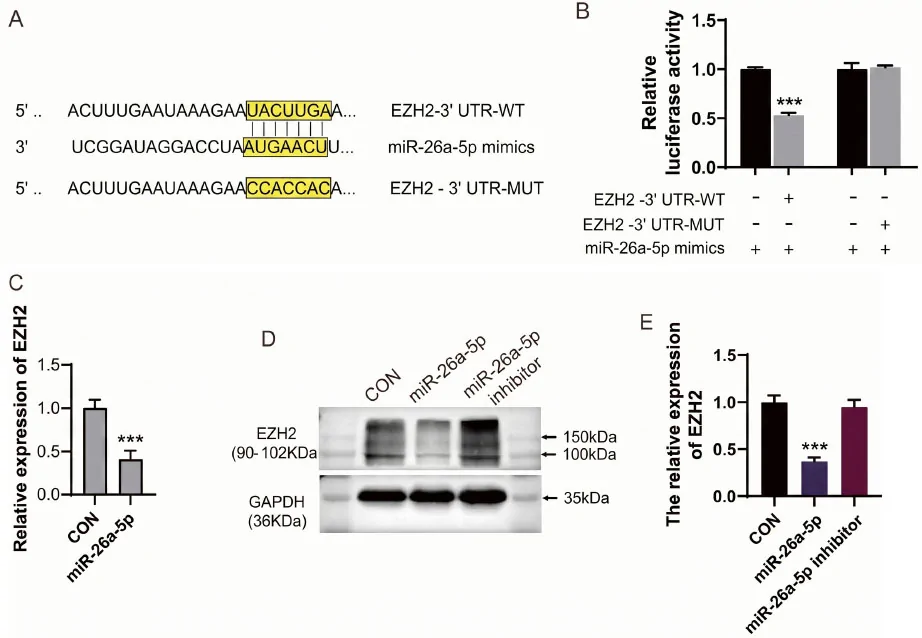
miR-26a-5p inhibited EZH2 expression through direct binding to EZH2. (**A**) Predicted binding sites between miR-26a-5p and EZH2. (**B**) The combination of miR-26a-5p and EZH2 was analyzed using a luciferase reporter assay. Cells were transfected with miR-26a-5p mimics and a wild /mutant of EZH2 plasmid. (**C**) HTR8 cells were transfected with miR-26a-5p mimics, and the mRNA level of EZH2 was detected using qPCR. (**D, E**) HTR8 cells were transfected with miR-26a-5p mimics or inhibitors, and the protein level of EZH2 was detected using a western blot.

**Fig. (4):**
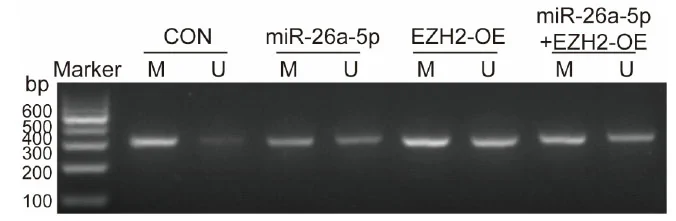
miR-26a-5p/EZH2 mediates WNT2 DNA methylation. HTR8 cells were transfected with miR-26a-5p, EZH2 expression plasmid (EZH2-OE), or co-transfected with miR-26a-5p and EZH2 expression plasmid (miR-26a-5p+EZH2-OE). Methylation of Wnt2 was detected using MSP.

**Fig. (5):**
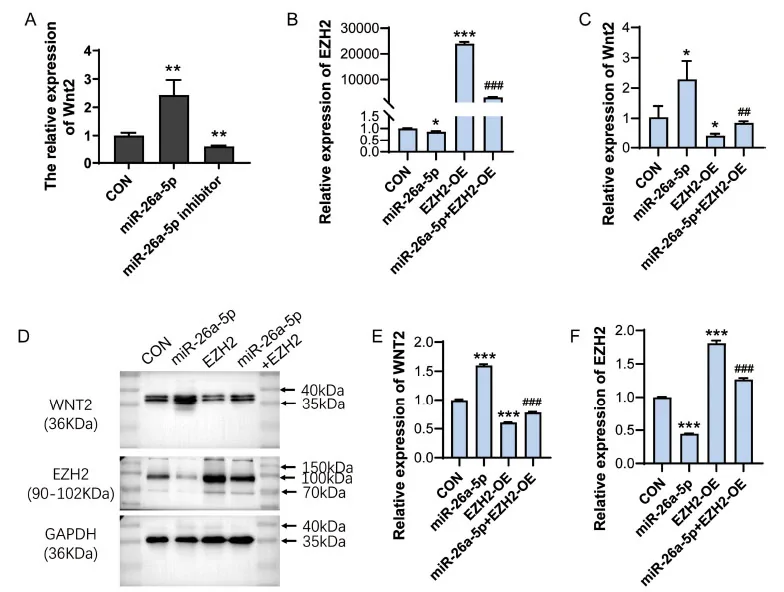
miR-26a-5p/EZH2 regulated the expression of Wnt2. (**A**) HTR8 cells were transfected with miR-26a-5p mimics or inhibitors. The mRNA level of Wnt2 was detected using qPCR. (**B**) HTR8 cells were transfected with miR-26a-5p, EZH2 expression plasmid (EZH2-OE), or co-transfected with miR-26a-5p and EZH2 expression plasmid (miR-26a-5p+EZH2-OE). EZH2 mRNA level was determined by qPCR. (**C**) Wnt2 mRNA level was determined by qPCR. (**D-F**) The protein levels of EZH2 and Wnt2 were detected using a western blot. * *P<*0.05 *vs.* CON, *** *P<*0.001 *vs.* CON, ## *P<*0.01 *vs.* EZH2-OE, ### *P<*0.001 *vs.* EZH2-OE.

## Data Availability

The data supporting the conclusions of this study are included in the manuscript.

## LIST OF ABBREVIATIONS

CCK-8: Cell Counting Kit-8
EOPE: early-Onset Preeclampsia
EZH2: Enhancer of Zeste 2 Polycomb Repressive Complex 2 Subunits
miRNAs: MicroRNAs
MSP: Methylation-Specific PCR
MUT: Mutant Type
PcG: Polycomb-Group
PE: Preeclampsia
qRT-PCR: Quantitative Real-Time PCR
WT: Wild Type

## References

[1] Al-Jameil N., Aziz Khan F., Fareed Khan M., Tabassum H. (2014). A brief overview of preeclampsia.. J. Clin. Med. Res..

[2] Yang Y., Le Ray I., Zhu J., Zhang J., Hua J., Reilly M. (2021). Preeclampsia prevalence, risk factors, and pregnancy outcomes in Sweden and China.. JAMA Netw. Open.

[3] Burton G.J., Redman C.W., Roberts J.M., Moffett A. (2019). Pre-eclampsia: pathophysiology and clinical implications.. BMJ.

[4] Saliminejad K., Khorram Khorshid H.R., Soleymani Fard S., Ghaffari S.H. (2019). An overview of microRNAs: Biology, functions, therapeutics, and analysis methods.. J. Cell. Physiol..

[5] Zhang Y., Lv L., Zheng R., Xie R., Yu Y., Liao H., Chen J., Zhang B. (2023). Transcriptionally regulated mir-26a-5p may act as brcaness in triple-negative breast cancer.. Breast Cancer Res..

[6] Kang L., Yang C., Wu H., Chen Q., Huang L., Li X., Tang H., Jiang Y. (2017). miR-26a-5p regulates TNRC6A expression and facilitates theca cell proliferation in chicken ovarian follicles.. DNA Cell Biol..

[7] Li M., Xiao Y., Liu M., Ning Q., Xiang Z., Zheng X., Tang S., Mo Z. (2022). MiR-26a-5p regulates proliferation, apoptosis, migration and invasion via inhibiting hydroxysteroid dehydrogenase like-2 in cervical cancer cell.. BMC Cancer.

[8] Lv Y., Lu C., Ji X., Miao Z., Long W., Ding H., Lv M. (2019). Roles of microRNAs in preeclampsia.. J. Cell. Physiol..

[9] Juárez-Barber E., Segura-Benítez M., Carbajo-García M.C., Bas-Rivas A., Faus A., Vidal C., Giles J., Labarta E., Pellicer A., Cervelló I., Ferrero H. (2023). Extracellular vesicles secreted by adenomyosis endometrial organoids contain miRNAs involved in embryo implantation and pregnancy.. Reprod. Biomed. Online.

[10] Li Q., Liu X., Liu W., Zhang Y., Wu M., Chen Z., Zhao Y., Zou L. (2021). MALAT1 sponges miR-26a and miR-26b to regulate endothelial cell angiogenesis via PFKFB3-driven glycolysis in early-onset preeclampsia.. Mol. Ther. Nucleic Acids.

[11] Katoh M. (2003). WNT2 and human gastrointestinal cancer (review).. Int. J. Mol. Med..

[12] Chen G., Wang C., Wang J., Yin S., Gao H., Xiang L., Liu H., Xiong Y., Wang P., Zhu X., Yang L., Zhang R. (2016). Antiosteoporotic effect of icariin in ovariectomized rats is mediated via the Wnt/β-catenin pathway.. Exp. Ther. Med..

[13] Zhang Z., Zhang L., Zhang L., Jia L., Wang P., Gao Y. (2013). Association of Wnt2 and sFRP4 expression in the third trimester placenta in women with severe preeclampsia.. Reprod. Sci..

[14] McGeary S.E., Lin K.S., Shi C.Y., Pham T.M., Bisaria N., Kelley G.M., Bartel D.P. (2019). The biochemical basis of microRNA targeting efficacy.. Science.

[15] Wang X.W., Yang S.G., Hu M.W., Wang R.Y., Zhang C., Kosanam A.R., Ochuba A.J., Jiang J.J., Luo X., Guan Y., Qian J., Liu C.M., Zhou F.Q. (2023). Histone methyltransferase Ezh2 coordinates mammalian axon regeneration via regulation of key regenerative pathways.. J. Clin. Invest..

[16] Zhang L., Leng M., Li Y., Yuan Y., Yang B., Li Y., Yuan E., Shi W., Yan S., Cui S. (2019). Altered DNA methylation and transcription of WNT2 and DKK1 genes in placentas associated with early-onset preeclampsia.. Clin. Chim. Acta.

[17] Ye Y., Tang Y., Xiong Y., Feng L., Li X. (2019). Bisphenol A exposure alters placentation and causes preeclampsia-like features in pregnant mice involved in reprogramming of DNA methylation of WNT2.. FASEB J..

[18] Frazier S., McBride M.W., Mulvana H., Graham D. (2020). From animal models to patients: the role of placental microRNAs, miR-210, miR-126, and miR-148a/152 in preeclampsia.. Clin. Sci. (Lond.).

[19] Illarionov R.A., Maltseva A.R., Pachuliia O.V., Postnikova T.B., Vashukova E.S., Popova A.K., Nasykhova Y.A., Bespalova O.N., Glotov A.S. (2024). Urinary miRNA expression in pre-eclampsia during early and mid-pregnancy.. Noncoding RNA.

[20] Chatterjee B., Thakur S.S. (2024). miRNA–protein–metabolite interaction network reveals the regulatory network and players of pregnancy regulation in dairy cows.. Front. Cell Dev. Biol..

[21] Zhou J., Zhao Y., An P., Zhao H., Li X., Xiong Y. (2023). Hsa_circ_0002348 regulates trophoblast proliferation and apoptosis through miR-126-3p/BAK1 axis in preeclampsia.. J. Transl. Med..

[22] Wu S., Li Q., Liu X., Huang H., Wang G., Zhang C., Meng Y., Yang W. (2023). Placental exosomal miR-125b triggered endothelial barrier injury in preeclampsia.. Placenta.

[23] Lu J., Getz G., Miska E.A., Alvarez-Saavedra E., Lamb J., Peck D., Sweet-Cordero A., Ebert B.L., Mak R.H., Ferrando A.A., Downing J.R., Jacks T., Horvitz H.R., Golub T.R. (2005). MicroRNA expression profiles classify human cancers.. Nature.

[24] Bian J., Ge W., Jiang Z. (2024). miR-26a-5p attenuates oxidative stress and inflammation in diabetic retinopathy through the USP14/NF-κB signaling pathway.. J. Ophthalmol..

[25] Li J., Pang D., Zhou L., Ouyang H., Tian Y., Yu H. (2024). miR-26a-5p inhibits the proliferation of psoriasis-like keratinocytes in vitro and in vivo by dual interference with the CDC6/CCNE1 axis.. Aging (Albany NY).

[26] Tang L., Wang W., Tang Q., Wang L. (2024). The molecular mechanism of MiR-26a-5p regulates autophagy and activates NLRP3 inflammasome to mediate cardiomyocyte hypertrophy.. BMC Cardiovasc. Disord..

[27] Yang Y., Xi L., Ma Y., Zhu X., Chen R., Luan L., Yan J., An R. (2019). The lncRNA small nucleolar RNA host gene 5 regulates trophoblast cell proliferation, invasion, and migration via modulating miR‐26a‐5p/N‐cadherin axis.. J. Cell. Biochem..

[28] Potiris A., Stavros S., Zouganeli I., Machairiotis N., Drakaki E., Zikopoulos A., Anagnostaki I., Zachariou A., Gerede A., Domali E., Drakakis P. (2024). Investigating the imperative role of micrornas expression in human embryo implantation: A narrative review based on recent evidence.. Biomedicines.

[29] Szuszkiewicz J., Nitkiewicz A., Drzewiecka K., Kaczmarek M.M. (2022). miR-26a-5p and miR-125b-5p affect trophoblast genes and cell functions important during early pregnancy.. Biol. Reprod..

[30] Hill M., Tran N. (2021). miRNA interplay: mechanisms and consequences in cancer.. Dis. Model. Mech..

[31] Liu H., Tang T. (2023). Pan-cancer genetic analysis of disulfidptosis-related gene set.. Cancer Genet..

[32] Fonseca Á.Y.G., González-Giraldo Y., Santos J.G., Aristizábal-Pachón A.F. (2023). The hsa-miR-516a-5p and hsa-miR-516b-5p microRNAs reduce the migration and invasion on T98G glioblastoma cell line.. Cancer Genet..

[33] Liu Y., Ma Y. (2017). Promoter methylation status of WNT2 in placenta from patients with preeclampsia.. Med. Sci. Monit..

[34] Wang G., Zhang Z., Chen C., Zhang Y., Zhang C. (2016). Dysfunction of WNT4/WNT5A in deciduas.. J. Hypertens..

[35] Li S., Li N., Li B., Zhu L., Xu T., Wang L., Zhang J., Kong F. (2022). CircHIPK3 promotes proliferation and metastasis of villous trophoblasts through miR-30a-3p/Wnt2 axis.. J. Genet..

[36] Zhao Y.H., Liu Y.L., Fei K.L., Li P. (2020). Long non-coding RNA HOTAIR modulates the progression of preeclampsia through inhibiting miR-106 in an EZH2-dependent manner.. Life Sci..

[37] Qian X., Zhang Y. (2022). EZH2 enhances proliferation and migration of trophoblast cell lines by blocking GADD45A-mediated p38/MAPK signaling pathway.. Bioengineered.

[38] Ningxia Z., Shuyan L., Zhongjing S., Ling C., Tianzhong M., Lifeng W., Yan Y., Leili L., Xiancai C., Haibin C. (2011). The expression pattern of polycomb group protein Ezh2 during mouse embryogenesis.. Anat. Rec. (Hoboken).

[39] Li B., Xia Y., Mei S., Ye Z., Song B., Yan X., Lin F., Rao T., Yu W., Mei C., Lv J., Wu M., Mao Z., Zhou X., Cheng F. (2023). Histone H3K27 methyltransferase EZH2 regulates apoptotic and inflammatory responses in sepsis-induced AKI.. Theranostics.

[40] Shang Y., Wu S., Li S., Qin X., Chen J., Ding J., Yang J. (2022). Downregulation of EZH2 in trophoblasts induces decidual m1 macrophage polarization: A potential cause of recurrent spontaneous abortion.. Reprod. Sci..

[41] Yeung K.R., Chiu C.L., Pidsley R., Makris A., Hennessy A., Lind J.M. (2016). DNA methylation profiles in preeclampsia and healthy control placentas.. Am. J. Physiol. Heart Circ. Physiol..

[42] Yuen R.K.C., Avila L., Peñaherrera M.S., von Dadelszen P., Lefebvre L., Kobor M.S., Robinson W.P. (2009). Human placental-specific epipolymorphism and its association with adverse pregnancy outcomes.. PLoS One.

[43] Yu M., Ting D.T., Stott S.L., Wittner B.S., Ozsolak F., Paul S., Ciciliano J.C., Smas M.E., Winokur D., Gilman A.J., Ulman M.J., Xega K., Contino G., Alagesan B., Brannigan B.W., Milos P.M., Ryan D.P., Sequist L.V., Bardeesy N., Ramaswamy S., Toner M., Maheswaran S., Haber D.A. (2012). RNA sequencing of pancreatic circulating tumour cells implicates WNT signalling in metastasis.. Nature.

[44] Liu Y., Huang J., Yu N., Wei S., Liu Z. (2020). Involvement of WNT2 in trophoblast cell behavior in preeclampsia development.. Cell Cycle.

[45] Li N., Li S., Wang Y., Wang J., Wang K., Liu X., Li Y., Liu J. (2017). Decreased expression of WNT2 in villi of unexplained recurrent spontaneous abortion patients may cause trophoblast cell dysfunction via downregulated Wnt/β-catenin signaling pathway.. Cell Biol. Int..

[46] Ferreira J.C., Choufani S., Grafodatskaya D., Butcher D.T., Zhao C., Chitayat D., Shuman C., Kingdom J., Keating S., Weksberg R. (2011). WNT2 promoter methylation in human placenta is associated with low birthweight percentile in the neonate.. Epigenetics.

